# Synthetic cell research: Is technical progress leaving theoretical and epistemological investigations one step behind?

**DOI:** 10.3389/frobt.2023.1143196

**Published:** 2023-03-23

**Authors:** Pasquale Stano, Luisa Damiano

**Affiliations:** ^1^ Department of Biological and Environmental Sciences and Technologies (DiSTeBA), University of Salento, Lecce, Italy; ^2^ RG-ESA (Research Group on the Epistemology of the Sciences of the Artificial), Libera Università di Lingue e Comunicazione (IULM), Milan, Italy

**Keywords:** autopoiesis, artificial or synthetic models, cognition, cybernetics, epistemology of the sciences of the artificial, philosophy of science, synthetic biology, synthetic cells

## Abstract

Advancements in the research on so-called “synthetic (artificial) cells” have been mainly characterized by an important acceleration in all sorts of experimental approaches, providing a growing amount of knowledge and techniques that will shape future successful developments. Synthetic cell technology, indeed, shows potential in driving a revolution in science and technology. On the other hand, theoretical and epistemological investigations related to what synthetic cells “are,” how they behave, and what their role is in generating knowledge have not received sufficient attention. Open questions about these less explored subjects range from the analysis of the organizational theories applied to synthetic cells to the study of the “relevance” of synthetic cells as scientific tools to investigate life and cognition; and from the recognition and the cultural reappraisal of cybernetic inheritance in synthetic biology to the need for developing concepts on synthetic cells and to the exploration, in a novel perspective, of information theories, complexity, and artificial intelligence applied in this novel field. In these contributions, we will briefly sketch some crucial aspects related to the aforementioned issues, based on our ongoing studies. An important take-home message will result: together with their impactful experimental results and potential applications, synthetic cells can play a major role in the exploration of theoretical questions as well.

## 1 Introduction

In recent years, the research on so-called “synthetic (artificial) cells” (SCs) has been characterized by a surprisingly strong momentum. The community of practitioners has grown significantly, also thanks to coordination initiatives like the MaxSynBio consortium in Germany, the BaSyC (Building a Synthetic Cell) project in the Netherlands, the European Synthetic Cell Initiative (SynCellEU), the Build-a-Cell community in the US, the fabriCELL project in the UK, Japanese programs such as CREST-PRESTO, and the research promoted by the Japanese Society for Cell Synthesis Research (Luisi, 2002; Salehi-Reyhani et al., 2017; Schwille et al., 2018; Stano, 2019; Staufer et al., 2021; Guindani et al., 2022).

A very impressive acceleration in experimental studies on the construction of SCs of different types can be identified just by looking at the number and quality of articles published on these subjects, very often in high impact-factor journals. This intense phase of research will probably attract many young and motivated scientists, who will pursue advanced studies on these “bottom-up” synthetic biology (SB) approaches in the upcoming years. Moreover, the combination of several strategies has produced a quite diversified research arena that literally shows the lively developments and the general enthusiasm around this topic—which is probably one of the most exciting among novel technologies. “SC technology,” indeed, does not resemble anything previously existing and shows potential in driving a revolution in future basic and applied sciences.^1^

Despite this impressive technical progress, important theoretical and epistemological issues have not received significant attention yet. We refer to fundamental questions such as 1) what SCs really are, i.e., what they represent in the context of artificial systems; 2) how their structure, properties, and behavior should be interpreted and with respect to which theoretical framework; and 3) what their role is in advancing scientific knowledge (what sort of knowledge SC research can generate). In this article, we are going to briefly consider some theoretical and epistemological issues related to SCs, selected among a list of subjects we consider particularly relevant to boost theoretical progress in this field (Table 1).

**TABLE 1:** List of theoretical and epistemological SC-related issues currently investigated by the authors.

Key notion	Details	Reference to our previous work
*Cybernetic Inheritance*	Synthetic biology and SCs as a late product (and a reappraisal) of cybernetics (1st vs. 2nd order) and systems theory	Damiano and Stano (2018)
		Damiano and Stano (2023)
*Organizational Theories of Living Systems*	Understanding and distinguishing types of relevance with respect to theories of reference, also focusing on the organization of the living (e.g., autopoiesis, chemoton, (*M*,*R*)-systems)	Damiano and Stano (2020)
		Damiano and Stano (2023)
*Cognitive Sciences and Explorative SB-AI*	Exploration of the emergence of minimal biological cognition, self, mind-like characteristics	Damiano and Stano (2018)
		Damiano and Stano (2021a)
		Damiano and Stano (2021b)
*Applicative SB-AI*	Possibility of using SC research to contribute to AI (and *vice versa*), e.g., by implanting chemical AI devices in synthetic cells	Gentili and Stano (2022)
		Stano (2022a)
		Stano et al. (2022)
*Complexity*	Definition of the complexity of natural and synthetic cell complexity, and possible ranking of synthetic cells	Damiano and Stano (2020)
		Gentili and Stano (2023)
*Information Theories*	Application of syntactic (C. Shannon) vs. semantic (D. M. MacKay, G. Bateson) theories, role of (cyber)semiotics (D. Nauta), and emergence of meaning	Magarini and Stano (2021)
		Stano (2022b)
		Ruzzante et al. (2023)

In particular, in this Perspective, we will briefly sketch what we believe are crucial aspects of the first three entries in Table 1 and present some preliminary ideas based on our already-published and ongoing studies. This will be an opportunity to present a research path that conjugates chemistry, SB, and philosophy of science questions. An important finding will be observed: together with their impactful experimental results and potential applications, SCs (and, more generally, SB) can play a major role in the exploration of theoretical and epistemological questions.

## 2 The inheritance from cybernetics

Our path of cross-fertilization between technical and theoretical–epistemological aspects of SC research is grounded in the legacy left by cybernetics for this emerging area and, in particular, in the cybernetic foundations of the synthetic modeling of life and cognition. Conceiving SCs as cellular models is indeed tantamount to considering SCs as scientific tools (perhaps “the” scientific tools *par excellence*) for investigating the generative mechanisms and the emergence of life at the minimal complexity level, corresponding to simple unicellular organisms. However, as we will clarify in the following paragraphs, cognition is a property closely related, or even coincident, with the property of being alive (e.g., Bich and Damiano, 2012; Damiano and Stano, 2018). It is not surprising, then, that life and cognition have been envisioned as interwoven targets by scientists and philosophers.

As early as 1943, a series of inaugural works from pioneers of cybernetics—specifically, McCulloch and Pitts (1943), Craik (1943), Rosenblueth, Wiener and Bigelow (1943)—proposed epistemological and theoretical frameworks to ground the exploration of biological and cognitive processes *via* construction and experimental exploration of artificial systems—systems “made by man rather than nature” (Langton, 1989)—functioning as material models of these target biological processes. These authors’ groundbreaking work is not limited to the introduction of the “synthetic method,” i.e., the “understanding-by-building” approach, as a strategy to study experimentally the mechanisms underlying life and cognition not only in their components (analytic method) but also “in their functioning” (synthetic method). As particularly evident in the 1943 McCulloch–Pitts and Rosenblueth–Wiener–Bigelow articles, cybernetics has also associated synthetic modeling with the possibility, for scientific research, of releasing cognition from the “ghostly” status it had assumed in modern (Cartesian) science and reintegrating it among the processes explorable through experimental and quantitative research. In other words: to overcome the mind–body dualism characterizing the Cartesian, or modern, tradition of science and make operational a series of *avant-garde* theses that today characterize the embodied front of cognitive sciences and artificial intelligence (AI). In a nutshell, they can be summarized in the following two claims: 1) the biological body plays a significant role in natural cognitive processes, and thus, to study cognition based on the synthetic method, effective ways of modeling synthetically body dynamics and interactions are needed. 2) Given the biochemical nature of the body, the synthetic modeling of natural cognitive processes is likely to be more successful when based on biochemical techniques—i.e., wetware models of bodily processes and interactions.

“If an engineer were to design a robot, roughly similar in behavior to an animal organism, he would not attempt at present to make it out of proteins and other colloids. He would probably build it out of metallic parts, some dielectrics and many vacuum tubes. The movement of the robot could readily be much faster and more powerful than those of the original organism. Learning and memory, however, would be quite rudimentary. In future years, as the knowledge of colloids and protein increases, future engineers may attempt the design of robots not only with a behavior, but also with a structure similar to that of a mammal.” (Rosenblueth et al., 1943, p. 23).

This is the cybernetic legacy that, more than 50 years later, is committing SB to engage in the areas of biology and scientific AI. Since the era of cybernetics, SB has been considered by many scholars as the most promising candidate for approaching at the experimental levels the exploration of life and cognition by means of artificial models. Wetware approaches, typical of SB, promise to constitute a third dimension complementing, in a particularly relevant manner, hardware (robotic) and software (AI) approaches, thus forming with them a plural “science of the artificial” (Cordeschi, 2002; Damiano et al., 2011). However, the possibility of concretely offering SB this candidacy, in addition to technical advances, requires addressing a series of open theoretical and epistemological questions.

The following two sections of this short article intend to introduce a few of these questions and the related research lines that we have opened to tackle them.

## 3 The problem of the relevance of synthetic (SB/SC) models

SB’s transition to the status of an accepted science of life and cognition does not depend only on the soundness of technical solutions in modeling biological and cognitive processes. It also depends primarily, on the possibility, for SB, to address effectively the epistemological open questions concerning the synthetic modeling of life and cognition, which, if left unanswered, threaten its acceptance among the methodological strategies that the scientific community recognizes capable of producing valid insights. Among these issues, a particularly critical issue questions the *relevance* of synthetic models, understood as the contribution(s) that they can make to the scientific understanding of their target processes.^2^ The problem is particularly critical since synthetic models often appear to have a merely “imitative” value, whose significance for the advancement of scientific knowledge of life and cognition is uncertain. Furthermore, the current evaluation of synthetic models is often polarized in the sterile alternative between, on one hand, mere behavioral imitation and, on the other hand, full reproduction of target processes, whose relevance is not less problematic. Indeed, although models based on imitation of behaviors of the target systems—i.e., simple functional equivalence—are *underdetermined*, models that would reproduce in detail all the physicochemical characteristics of the target systems would be *overdetermined* as their realism would involve the inclusion of physical and functional properties, obscuring, instead of clarifying the mechanism underlying life and cognition.

**FIGURE 1:**
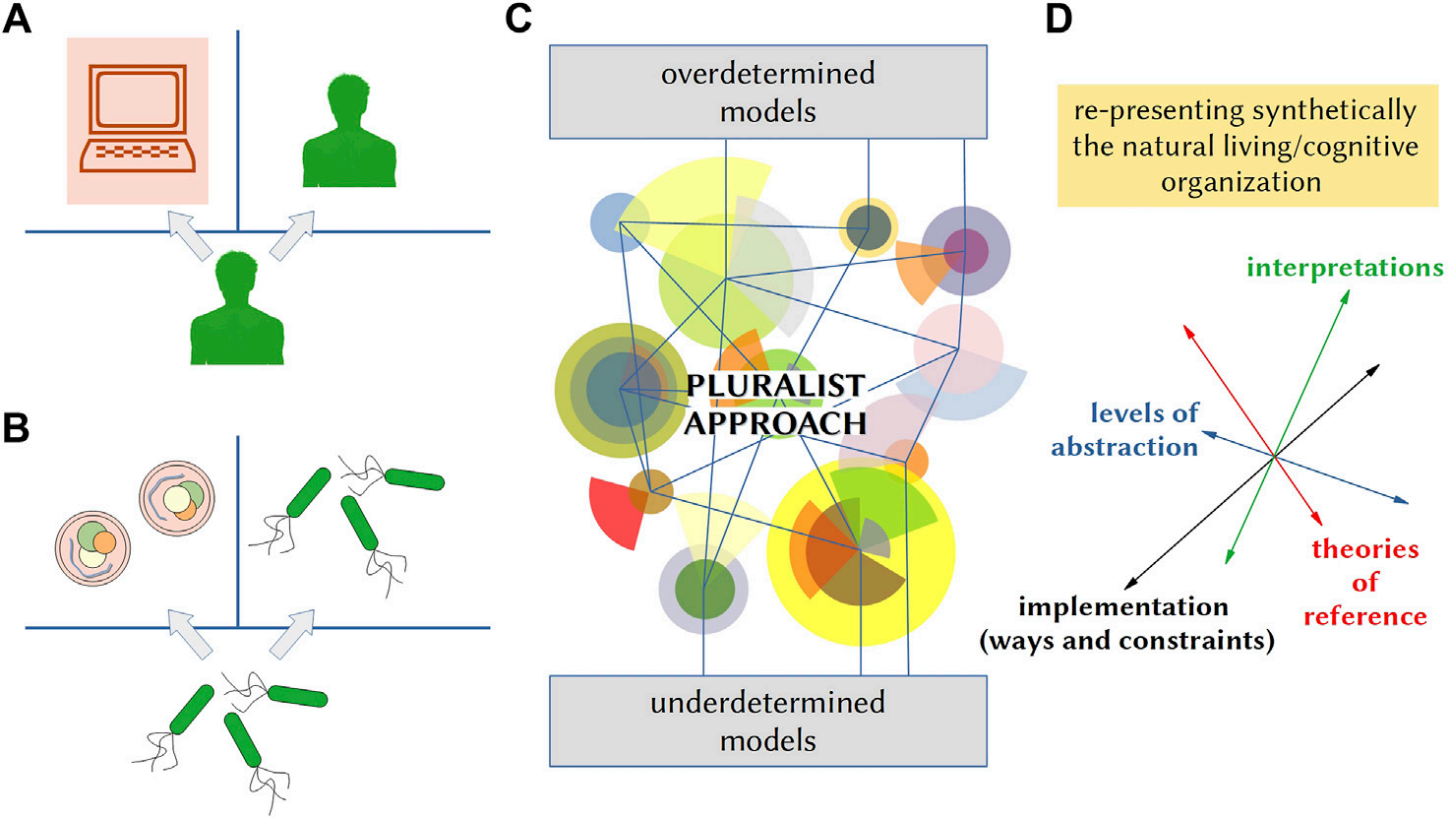
Problem of the relevance of synthetic (SB/SC) models. **(A)** Schematic representation of the classical version of the Turing test (Turing, 1950), also known as the imitation game. The test was devised to bypass the question “can a machine think?” or “what is intelligence?” and substitute it with an operational definition. A human interrogator blindly interacts with a computer or with another human. The machine behavior is defined “intelligent” when the interrogator is not able to distinguish it from a human. The machine “imitates” human intelligence. **(B)** Schematic representation of the Turing test in a SB scenario (Cronin et al., 2006). Living cells (e.g., bacteria) and SCs have substituted humans and computers. Chemical signaling or other kinds of interactions substitute the written dialogs (questions/answers) on which the original Turing test is based. By analogy, the test can be considered a tool to bypass the question “can a man-made chemical system be alive/cognitive?” or “what is life/cognition?,” providing an operational definition. A synthetic model of living/cognitive systems is alive/cognitive when living cells are not able to distinguish it from other living cells (more generally: when the synthetic model is behaviorally equivalent, for living cells, to what they perceive and dynamically interact with as their own environment). **(C)** Pictographic representation of the wide research space opened between underdetermined and overdetermined models, when a pluralist approach to the synthetic modeling of life and cognition is adopted. **(D)** Latter approach intends to overcome mere imitative modeling of life and cognition and proposes the scientific community to attempt at representing the biological/cognitive organizations synthetically by exploring a variety of theories of reference, diverse interpretations of these theories, and multiple options available with regard to the levels of abstraction, as well as the ways (and related constraints) of their implementation.

This epistemological view, diffused in the debate since the era of cybernetics, has been mostly neglected in the context of synthetic modeling, where, since the 1950s, the most popular tool to assess the value of models is the Turing test (Turing, 1950), which focuses on their ability of imitating the target systems’ *manifest behavior*. Despite many critiques and reformulations, for which we refer to the literature, this test still constitutes a paradigmatic reference for evaluating the relevance of synthetic models, not only in AI but also in the field of explorative SB (Figures 1A,B), where it has been the basis of the first attempts at assessing the life-likeness of SCs (Cronin et al., 2006; Lentini et al., 2017; for a commentary, see Damiano and Stano, 2020). In our view, to effectively address the problem of the relevance of synthetic models, we need new criteria of relevance capable of overcoming both the traditional exclusive attention to imitation and the diffused polarization of the assessment between mere imitation and full reproduction of the target processes.

To fill this gap, we have undertaken an epistemological inquiry into the relevance of synthetic models. This research builds on elements of cybernetic and autopoietic epistemology to determine criteria to assess the different forms of relevance that (hardware, software, and wetware) models can have for the scientific understanding of life and cognition. As mentioned, the aim is the clarification of the contribution that SB can produce to advance scientific knowledge on life and cognition. Let us summarize here the essential elements that emerged from our exploration, a full discussion of which can be found elsewhere (Damiano and Stano, *Artificial Life*, in press).

Our work generated two relevance criteria for synthetic models which, to overcome the classical imitation paradigm and the related pure imitation/complete reproduction polarization, emphasize the importance of focusing the synthetic exploration of life and cognition on the *organization* underlying the target processes. This view, in our research, is associated with a pluralist perspective on synthetic modeling, according to which, to recreate synthetically the organizational mechanisms that in nature are responsible for producing a natural process, does not mean to reproduce “the real thing,” since this would require, from an epistemological viewpoint, the availability of a definitive, exhaustive, univocally interpretable, and perfectly implementable theory of the biological and cognitive organization—something beyond the reach of scientific research. In our view, any attempts at reproducing the organization of living and/or cognitive processes have to be based on one or more selections among a multiplicity of options since every target theory of biological and/or cognitive organization can be interpreted in different ways, each of its interpretations can be realized synthetically at a variety of different levels of abstraction, and each of these synthetic realizations, in order to be produced, requires addressing specific implementative constraints, which can be tackled in different ways.

This is the core of the pluralist approach to the synthetic modeling of life and cognition, which opens up a generative research space between underdetermined and overdetermined models (Figure 1C). Indeed, this approach engages the scientific community in implementing a variety of theories of biological/cognitive organization and, with regard to each of them, in exploring a variety of different ways of implementation, based on diverse interpretations of the theory of reference and the multiple options available with regard to the level of abstraction defining the synthetic realization (Figure 1D). In our perspective, scientifically, this approach is more generative than any attempts at overcoming imitation by trying to reproduce “the real thing”, as it is likely to generate a wide multiplicity of valuable insights, in line with the Langtonian ambition of creating a synthetic science of life and cognition as they are and as they “could be” (Langton, 1989).

## 4 The problem of representing the organizational complexity of life and cognition synthetically

Any attempt to build organizationally relevant synthetic models of biological and/or cognitive processes requires first choosing a theory of reference that offers a scientific description of the organization of life and/or cognition.

Among the organizational theories accessible today, our choice fell on autopoietic cognitive biology (Maturana and Varela, 1973) since this theory, on one hand, thematizes a profound continuity between life and cognition and, on the other hand, proposes a description of the biological and cognitive organization at the level of the minimal living unit. On this basis, this theory offers SB—and, in particular, SC research—a key role in the scientific understanding of cognitive processes. Indeed, autopoiesis generates the theoretical grounds to explore experimentally the controversial thesis that “life, as a process, is a process of cognition” (Maturana, 1969), and in this way, it proposes the experimental option of studying the threshold of minimal life and minimal cognition, as well as their relationship, by physically constructing wetware models of living/cognitive systems characterized by minimal complexity. This thesis, while being controversial, is extremely interesting for the purpose of de-anthropocentering the traditional philosophical and scientific views of cognitive processes. The related wetware experimental approach, proposing a wet version of the “understanding-by-building” methodology, relies on chemical and/or biochemical elements and SC techniques.

However, the design and implementation of autopoietic systems in the laboratory involves, in addition to practical difficulties, a series of conceptual problems. The reason is that, even at the simplest level, autopoietic systems are characterized by an *organizational closure.* This theoretical notion indicates that the undergoing chemical processes and transformations need to be linked to each other based on a circular/reticular causality. Moreover, the network of transformations designed to constitute an autopoietic system, in order to be considered cognitive, has to be able to perceive environmental perturbations and to cope with some of them, at least, which means to self-regulate successfully, maintaining its own functional coherence and the underlying reticular organization.

Biological autopoietic systems have structures shaped by evolution, whereby the environment has had a participative, co-constructive role. The structure of an autopoietic system is somehow a map of its own history of structural coupling with the environment, which may include other autopoietic systems. In other words, the system embodies, in its peculiar realization and dynamics, “semantic information” about its world, which it co-created through interaction with its niche (Nauta, 1972; Varela, 1979). Such a system indeed learns to react to recurrent perturbations in its environment by associating them with endogenous patterns of self-regulation, i.e., endogenous operational meanings that define in what ways the system compensates for these alterations and maintains itself in the related perturbative external conditions. For example, a certain environmental event linked to a signaling pathway activates a certain gene, *a*, and not another gene, *b*, because for the system, only this specific route serves its intrinsic goal of self-maintenance and not another. This route is the operational meaning that the system associates with the related environmental event, based on its (phylogenetic and ontogenetic) history of coupling with its environment.

Therefore, although the technical issue behind the construction of artificial autopoietic systems refers to the practical possibility of designing and constructing such systems (not discussed here), the theoretical question is subtler and refers to the mechanisms of the generation of meaning for these system. In the case of hypothetical autopoietic–and thus cognitive–SCs, where do their meanings come from? Whether or not SCs are built by using biomacromolecules (to closely mimic biological cells), or by using allegedly primitive molecules (to mimic primitive cells), or by using fully artificial molecules (to produce authentic “artificial” cells), or by employing any sort of hybrid approach, the SC structure is ultimately devised by an experimenter. The experimenter decides, *a priori*, not only the SC structure (intended as the set of reactions, their topology, and dynamics) but also the environment into which SCs are embedded. Technical difficulties translate into simplified—often oversimplified—versions of the target system, while the definition of a “stiff” SC/environment super-system sacrifices the very important moment of meaning generation, which becomes possible only when plastic behavior is allowed. In this respect, it seems that the design of chemical systems more apt to adaptive behavior, plasticity, and easier endogenous reconfiguration is more promising than the design of systems based on the predictable behavior of complex biomacromolecules^3^. The latter will be performing more in terms of reproducing a series of cell-like behaviors in a programmable manner but probably only partially appropriate to reproduce cognitive features and the emergence of meaning (the question, however, is open to discussion). The sought scenario is somehow resonant with the scientific work realized on the synthetic modeling of cognition by Gordon Pask, another major contributor to (second-order) cybernetics who investigated rudimentary forms of electrochemical systems with the ability to adaptively construct their own sensors, thereby choosing the relationship between their internal states and the world at large (Pask, 1959; Cariani, 1993). Systems chemistry, a recently developed field of chemistry where these sorts of phenomena find a proper collocation, can ally with SB to provide a frontier platform to address these critical theoretical issues (Ruiz-Mirazo et al., 2014; Ashkenasy et al., 2017; Čejková and Cartwright, 2022).

## 5 Concluding remarks

The enthusiasm born around SB, and in particular around the construction of SCs, has several roots. In addition to the rapid technical development of the field, driven by an original combination of microcompartment technology and microfluidics, cell-free systems, and numerical modeling, which promise innovative contributions to novel biotechnologies, there are philosophical and epistemological scientific interests. They are motivated by the recent actualization of the possibility—prefigured in the cybernetic era—of studying cognition through the construction and experimental exploration of artificial systems capable of reproducing the phenomenological and organizational aspects of biological systems. Among the several open questions on the potential role of SB and SC in the epistemology of the science(s) of the artificial (for example, see Table 1), here, we have briefly discussed questions related to, on one hand, overcoming the imitation paradigm and the polarization ‘mere imitation/full reproduction’ of the target processes, in the context of the synthetic modeling, and, on the other hand, representing synthetically the complexity of the organization underlying natural life and cognition. Furthermore, we promoted a pluralist approach to the synthetic modeling of life and cognition, which aims at making generative the workspace between underdetermined and overdetermined models of biological and cognitive processes by implementing a variety of theories of biological/cognitive organization, and, with regard to each of them, exploring different ways of synthetic realization, based on the Langton-inspired idea of a synthetic science of life and cognition *as they are and they could be*. However, in these few pages, we could only offer a schematic overview of these issues, and for their appropriate discussion, we must refer to other works (Table 1). The most relevant message that we intended to convey in this short article emphasizes the importance of bringing to the attention of the community not only the technical issues, but also the theoretical and epistemological issues underlying the involvement of SB and, in particular, of SC research in AI, as this is the only way to fully unfold the potential that they can express in this field.

## Data Availability

The original contributions presented in the study are included in the article/Supplementary Material; further inquiries can be directed to the corresponding authors.

## Footnotes

1 As suggested by a reviewer, we would like to spend a few words about the definition of “synthetic (or artificial) cell” and its use in the scientific literature. Currently, this expression is widely used to indicate many types of microcompartments, e.g., coacervates, liposomes, and droplets, generated in the laboratory, which host in their volume or on their surface a *rather small set* of compounds often borrowed from cellular biochemical pathways (such as the set of macromolecules that carry out the transcription–translation reactions, or the enzymes required to catalyze a series of sequential reactions, or small genetic circuits*.*). They are very simple and non-living cell-like structures, which can be best intended as rudimentary cytomimetic chemical systems. The resulting structures cannot be really compared to living cells, even when “minimal” living cells (actual or hypothetical) are considered. Studies in comparative genomics have shown, indeed, that the minimal genome still counts, in very permissive environmental conditions, 200–250 genes (Mushegian and Koonin, 1996; Luisi et al., 2002; Gil et al., 2004). Experiments with synthetic genomes have shown that a viable minimal living cell—called JCVI-syn3A—is based on 493 genes, of which 452 code for proteins and 38 for RNAs (Hutchison et al., 2016). It should be noted, moreover, that the research on SCs actually originated in connection with origin-of-life problems, attempting *to model* early “protocells,” which can be considered pre-cellular structures lying at the interface between life and non-life stages (Oberholzer et al., 1995; Szostak et al., 2001; Luisi et al., 2006). Current SC approaches and SC technology, however, are not always directed to address origin-of-life questions (or theoretical biology concepts such as autopoiesis, e.g., Luisi, 2003) but also target applied science, biotechnology, complex bioassays, and nanomedicine.

2 It is worth mentioning that the issue of relevance is not limited to wetware models but also affects software and hardware models. For the three forms of synthetic models, indeed, the relationship they currently have with their target processes, and thus their explanatory power, is still unclear (Damiano and Stano, *Artificial Life*, in press).

3 Here, we refer to those designs whereby complex biomacromolecules (e.g., enzymes) are employed mainly because of their efficiency due to the strong constraints they impose on chemical reactions, allowing the very act of “designing” SCs. Biomacromolecule activities are, however, not completely independent from their environment: in turn, they can be allosterically regulated by third parties (activators and inhibitors, which can be elements of the network as well) so that enzyme-based chemical networks also exhibit—in a certain sense—a variation in their structure (e.g., variation of chemical flows, resulting in a change of the relations of productions of components within the network).
